# Severity of Non-Alcoholic Fatty Liver Disease Assessed by FibroScan in Patients With and Without Ischemic Heart Disease: A Cross-Sectional Study

**DOI:** 10.7759/cureus.97927

**Published:** 2025-11-27

**Authors:** Kaleemullah Shaikh, Shahid Karim, Noman Ahmed, Abdul Basit, Nida Batool, Fahad Kaleem

**Affiliations:** 1 Department of Cardiology, Liaquat National Hospital, Karachi, PAK; 2 Department of Gastroenterology, Liaquat National Hospital, Karachi, PAK; 3 Department of Dentistry, Ziauddin University, Karachi, PAK

**Keywords:** fibroscan, ischemic heart disease, liver fibrosis, non-alcoholic fatty liver disease, steatosis

## Abstract

Background

Non-alcoholic fatty liver disease (NAFLD) is the most common chronic liver disease and is closely linked to metabolic risk factors that also predispose to ischemic heart disease (IHD). Evaluating the relationship between NAFLD severity and IHD may improve cardiovascular risk assessment.

Objective

To compare the severity of NAFLD, assessed by FibroScan, in patients with and without IHD.

Methods

A cross-sectional study was conducted at Liaquat National Hospital, Karachi (Sept 2023-Feb 2024). Adults with NAFLD confirmed by FibroScan were enrolled (n=300), including 100 IHD patients and 200 non-IHD controls. Anthropometric, biochemical, and FibroScan parameters (Controlled Attenuation Parameter (CAP) and Liver Stiffness Measurement (LSM)) were recorded. Between-group differences were analyzed using t-tests and Chi-square tests, and logistic regression was used to estimate associations, adjusting for age, sex, and BMI.

Results

Among the 300 patients (mean age 51.4 ± 8.5 years; 62.2% female), obesity (99.1%), diabetes (54.8%), and dyslipidemia (62.7%) were prevalent comorbidities. Mean FibroScan values did not differ significantly between IHD and control groups (7.2±2.05 vs 7.03±2.16 kPa, p=0.507). CAP scores were also comparable (273.3±65.0 vs 282.2±66.6 dB/m, p=0.276). Logistic regression showed mild CAP associated with higher IHD odds (OR 2.21, 95% CI 1.04-4.72, p=0.04), but no consistent associations were observed with fibrosis stage.

Conclusions

The severity of non-alcoholic fatty liver disease (NAFLD), assessed non-invasively by FibroScan, did not differ significantly between patients with and without ischemic heart disease (IHD). A borderline association between mild hepatic steatosis (CAP) and IHD was observed; however, this finding should be interpreted with caution, as it lacked a dose-response pattern and histologic (biopsy) confirmation. Given the cross-sectional design, the results indicate an association rather than causation, underscoring the need for longitudinal, biopsy-validated studies to better clarify the hepatic-cardiovascular relationship.

## Introduction

Non-alcoholic fatty liver disease (NAFLD) is now recognized as the most prevalent chronic liver condition globally, largely driven by the epidemics of obesity and metabolic syndrome [1]. A recent meta-analysis of 38 countries estimated the global prevalence at 30.2%, with regional rates ranging from 16.1% in Australia to 34% in South America [2]. Ehsan Amini-Salehi et al. conducted a meta-analysis including 78,001,755 participants, estimating the Asian NAFLD prevalence at 30.9% [3]. In parallel, ischemic heart disease (IHD) remains the leading cause of mortality worldwide, responsible for nearly nine million deaths annually [2]. The rising coexistence of NAFLD and IHD presents an urgent public health challenge, highlighting the need to better understand their interrelationship.

Evidence indicates that NAFLD and cardiovascular disease (CVD) share overlapping metabolic pathways and risk factors [4]. Often termed the hepatic manifestation of metabolic syndrome, NAFLD is closely linked to central obesity, dyslipidemia, insulin resistance, and hypertension. These factors create a pro-atherogenic state characterized by systemic inflammation, endothelial dysfunction, and atherogenic lipid profiles, all of which accelerate atherosclerosis [5]. Notably, even lean NAFLD patients face elevated cardiovascular risk, underscoring that NAFLD is not solely a byproduct of obesity. Cardiovascular complications, rather than liver failure, have emerged as the leading cause of death in patients with NAFLD, underscoring the systemic impact of this condition.

Large-scale epidemiologic studies further support NAFLD as an independent predictor of cardiovascular morbidity and mortality, including IHD. A meta-analysis involving nearly 5.8 million participants found that NAFLD confers a 45% higher risk of fatal or non-fatal CVD events, independent of age, sex, obesity, diabetes, or hypertension [6]. Moreover, disease severity matters; patients with advanced fibrosis or non-alcoholic steatohepatitis (NASH) have more than twice the risk of cardiovascular events compared to those with mild disease. Fibrosis, in particular, has emerged as the strongest histological predictor of adverse cardiovascular outcomes [7].

Liver biopsy remains the gold standard for staging NAFLD, but is invasive and unsuitable for routine practice [8-10]. Transient elastography (FibroScan) has therefore gained prominence as a non-invasive alternative, simultaneously measuring liver stiffness (LSM) as a marker of fibrosis and the controlled attenuation parameter (CAP) as an indicator of steatosis [11]. This tool demonstrates strong diagnostic accuracy for advanced fibrosis and significant steatosis, with guideline endorsements for risk stratification in clinical practice. Importantly, higher FibroScan-derived stiffness values have also been linked to elevated long-term atherosclerotic cardiovascular risk, even after adjusting for obesity and steatosis. 

However, despite growing global evidence, there is a paucity of data from South Asian populations comparing FibroScan-derived NAFLD severity between patients with and without ischemic heart disease (IHD). Most existing studies rely on ultrasound or biochemical definitions of fatty liver and originate from Western or East Asian cohorts. Given the high prevalence of both metabolic syndrome and premature cardiovascular events in South Asia, examining this relationship using quantitative elastography provides region-specific and clinically relevant insights. Therefore, the present study was designed to compare NAFLD severity, measured non-invasively by FibroScan, between patients with and without IHD, addressing this important evidence gap and exploring whether hepatic steatosis and fibrosis stages correlate with cardiovascular risk in a South Asian cohort.

## Materials and methods

Study design

This was a hospital-based cross-sectional study designed to compare the severity of non-alcoholic fatty liver disease (NAFLD) between patients with ischemic heart disease (IHD) and those without IHD, using FibroScan as the primary assessment tool.

Ethical considerations

The study protocol was reviewed and approved by the Institutional Review Board and Medical Ethics Committee of Liaquat National Hospital, Karachi. All participants provided written informed consent prior to enrollment, and the study was conducted in accordance with the principles of the Declaration of Helsinki.

Settings

Data were collected at the Department of Cardiology, Liaquat National Hospital, Karachi, Pakistan, between September 2023 and February 2024. The hospital is a tertiary care facility serving a large and diverse population, which increased the representativeness of the study sample.

Participants

Eligible participants were adults aged >20 years with non-alcoholic fatty liver disease (NAFLD) confirmed by FibroScan. NAFLD was defined as hepatic steatosis on FibroScan in the absence of competing causes of liver fat accumulation, including significant alcohol intake (>20 g/day in women or >30 g/day in men), which was assessed by structured interview. The case group comprised patients with ischemic heart disease (IHD), defined according to standardized diagnostic criteria: acute coronary syndrome or myocardial infarction verified by troponin elevation plus electrocardiographic changes and/or coronary angiography; chronic stable angina with objective ischemia on stress testing; or ≥50% luminal stenosis documented on angiography. Both prevalent and incident IHD diagnoses were eligible, provided they were confirmed in hospital records. Controls were NAFLD patients without IHD, recruited from the same catchment and time period under identical inclusion and exclusion criteria. Exclusion criteria included pregnancy, hepatitis B or C infection, radiologic evidence of cirrhosis on ultrasound or CT, prior splenectomy, incomplete lipid profile data, and excess alcohol consumption as defined above.

Recruitment and sampling

Participants were enrolled through consecutive sampling from the Department of Cardiology and associated outpatient clinics of Liaquat National Hospital during the study period. The case group consisted of adults with confirmed IHD meeting the diagnostic criteria described above, while the control group comprised patients without IHD who underwent FibroScan evaluation for fatty liver during the same period. Both groups were recruited under identical eligibility criteria to minimize selection bias. Controls were not individually matched on metabolic parameters (e.g., diabetes, hypertension, dyslipidemia) to permit independent assessment of these factors; however, frequency matching by age range and sex was maintained to ensure comparable demographic profiles.

Variables

The main exposure variable was the presence of ischemic heart disease, while the primary outcome variables were the severity of hepatic steatosis and fibrosis as measured by FibroScan. Potential confounders included age, sex, body mass index (BMI), diabetes, hypertension, dyslipidemia, smoking status, and other features of metabolic syndrome.

Data sources and measurement

Anthropometric measurements were obtained with participants wearing light clothing and no footwear. Body weight and height were measured to calculate BMI, and waist, hip, and neck circumferences were also recorded. Venous blood samples were collected after an overnight fast, and the lipid profile, including total cholesterol, triglycerides, HDL-C, and LDL-C, was analyzed using enzymatic colorimetric methods on the Roche Cobas 8000 modular analyzer. Dyslipidemia was defined as triglycerides ≥200 mg/dL, LDL-C ≥160 mg/dL, or HDL-C ≤40 mg/dL. Additional tests included HbA1c, liver enzymes, and renal function parameters. Hepatic steatosis and fibrosis were assessed by transient elastography (FibroScan, Echosens, Paris, France). Steatosis severity was categorized by controlled attenuation parameter (CAP) values into four stages: S0 (<238 dB/m, no steatosis), S1 (238-259 dB/m, mild), S2 (260-290 dB/m, moderate), and S3 (>290 dB/m, severe). Liver stiffness measurement (LSM) values were used to classify fibrosis into no, mild, significant, or advanced stages, according to standard cut-offs.

Study size

This study was planned to compare the severity of NAFLD between patients with and without IHD using FibroScan parameters (LSM and CAP). Sample size calculations were therefore based on the ability to detect differences in mean LSM between the two groups rather than on disease prevalence. With a 1:2 allocation (100 IHD vs 200 non-IHD), a two-sided α of 0.05, and 80% power, the achieved sample is able to detect mean between-group differences of approximately 0.69 kPa if the true standard deviation (SD) of LSM is 2.0 kPa, 0.82 kPa if the SD is 2.4 kPa, and 1.03 kPa if the SD is 3.0 kPa. Published NAFLD cohorts typically report LSM variability in the range of 2-3 kPa [12], while healthy Pakistani adults show narrower variability of about 0.6-1.1 kPa [13]. On this basis, the assumption of an SD of 2.0-3.0 kPa is both conservative and regionally plausible. Accordingly, the present study is sufficiently powered to detect between-group differences in LSM of roughly 0.7-1.0 kPa. Detecting a smaller difference, such as 0.5 kPa, would have required a substantially larger sample of more than 800 participants with the same 1:2 ratio. Logistic regression analyses were undertaken as exploratory assessments and were not used for a priori power determination.

Statistical methods

Data were analyzed using IBM SPSS Statistics for Windows, Version 27.0 (IBM Corp., Armonk, NY, USA). Between-group comparisons were performed using the independent t-test or the Wilcoxon rank-sum test for continuous variables and Pearson’s Chi-square or Fisher’s exact test for categorical variables. Logistic regression analysis was applied to estimate odds ratios (ORs) with 95% confidence intervals (CIs) for the association between NAFLD severity and IHD, adjusting for age, sex, and BMI. A two-sided p-value of less than 0.05 was considered statistically significant.

## Results

Table 1 presents baseline demographic, biochemical, and hepatic characteristics of patients with and without ischemic heart disease (IHD). Age, gender distribution, and anthropometric indices such as BMI, waist, hip, and neck circumference were comparable between groups. Most biochemical markers, including lipid profile, liver enzymes, bilirubin, creatinine, and blood counts, did not differ significantly. The only notable difference was lower HbA1c among IHD patients (6.36±1.3 vs. 6.8±1.4, p=0.009), indicating better glycemic control in this group. Hepatic assessment by FibroScan and CAP revealed similar mean values and distributions of steatosis and fibrosis stages across both groups. 

**Table 1:** Baseline demographic, clinical, biochemical, and hepatic characteristics of patients with and without ischemic heart disease. Data has been presented as mean ± SD, (n, %). An independent t-test was applied for continuous variables, and a Chi-square/Fisher's exact test was applied for categorical variables. *p-value≤0.05 is considered significant. CAP: controlled attenuation parameter, ALT: alanine transaminase, AST: aspartate transaminase, ALP: alkaline phosphatase. GGT: gamma-glutamyl transferase, TG: tri glycerides, LDL: low density lipoprotein, HDL: high density lipoprotein.

Parameters		Cases (n=100)	Controls (n=200)	t/ χ²	p-value
Age (years); mean ± SD		50.47 ± 8.42	51.94 ± 8.5	1.44	0.159
Age group (n, %)	≤45 years	35 (35)	51 (25.5)	2.94	0.086
	>45 years	65 (65)	149 (74.5)		
Gender (n, %)	Male	42 (42)	77 (38.5)	0.34	0.559
	Female	58 (58)	123 (61.5)		
Body mass index (kg/m²); mean ± SD		30.11 ± 3.47	30.76 ± 3.52	-1.52	0.13
Waist circumference (cm); mean ± SD		104.02 ± 8.03	103.68 ± 8.86	0.31	0.739
Hip circumference (cm); mean ± SD		108.14 ± 8.54	108.23 ± 9.31	-0.08	0.937
Neck circumference (cm); mean ± SD		43.3 ± 2.66	43.61 ± 3.67	-0.74	0.453
Obesity (n, %)	Yes	98 (98)	198 (99)	0.27	0.603
	No	2 (2)	2 (1.0)		
HDL (mg/dL); mean ± SD		37.71 ± 8.29	36.14 ± 7.67	1.59	0.113
LDL (mg/dL); mean ± SD		124.81 ± 17.8	125.3 ± 18.88	-0.22	0.829
Triglycerides (mg/dL); mean ± SD		134.26 ± 27.79	136.2 ± 30.62	-0.56	0.582
TLC(×10⁹/L); mean ± SD		8.55 ± 1.65	8.62 ± 1.44	-0.4	0.69
HbA1c (%); mean ± SD		6.36 ± 1.3	6.8 ± 1.4	-2.65	0.009*
Hemoglobin (g/dL); mean ± SD		11.83 ± 1.38	11.83 ± 1.6		0.967
Creatinine (mg/dL); mean ± SD		1.05 ± 0.36	1.04 ± 0.35	0.25	0.781
Total bilirubin (mg/dL); mean ± SD		1.16 ± 0.86	1.19 ± 0.85	-0.33	0.735
Direct bilirubin (mg/dL); mean ± SD		0.97 ± 1.23	0.89 ± 0.72	0.74	0.461
ALP (U/L); mean ± SD		86.69 ± 41.11	83.46 ± 26.01	0.82	0.407
GGT (U/L); mean ± SD		36.48 ± 15.66	37.83 ± 18.32	-0.63	0.529
Fasting RBS (mg/dL); mean ± SD		165.98 ± 65.08	167.36 ± 57.58	-0.19	0.852
AST (U/L); mean ± SD		39.3 ± 13.36	38.06 ± 13.06	0.86	0.441
Platelets (×10⁹/L); mean ± SD		300.8 ± 66.59	298.54 ± 66.96		0.782
ALT (U/L); mean ± SD		54.99 ± 17.91	51.1 ± 18.7	1.72	0.086
AST and ALT ratio; mean ± SD		0.78 ± 0.34	0.85 ± 0.39	-1.53	0.127
Serum cholesterol (mg/dL); mean ± SD		198.96 ± 58.26	198.56 ± 55.78	0.05	0.954
TG and HDL ratio; mean ± SD		3.82 ± 1.5	4.05 ± 1.68	1.72	0.251
	≤2	1 (1)	1 (0.5)	-	0.875
	2.1 to 4.0	68 (68)	130 (65)		
	4.1 to 6.0	19 (19)	42 (21)		
	>6.0	12 (12)	27 (13.5)		
LDL to HDL ratio		3.53 ± 1.19	3.72 ± 1.37	-1.21	0.225
	≤2.9	39 (39)	76 (38)	0.03	0.867
	>2.9	61 (61)	124 (62)		
Cholesterol to HDL ratio		5.77 ± 2.93	6.04 ± 3.11	-0.74	0.459
	≤3.5	12 (12)	25 (12.5)	0.12	0.725
	3.51 to 5.0	51 (51)	89 (44.5)		
	5.1 to 9.0	22 (22)	54 (27)		
	>9.0	15 (15)	32 (16)		
Hepatic steatosis score	0	15 (15)	52 (26)	5.75	0.104
	1	20 (20)	28 (14)		
	2	2 (2)	7 (3.5)		
	3	63 (63)	113 (56.5)		
Steatosis (n, %)	Yes	65 (65)	128 (64)	0.03	0.865
	No	35 (35)	72 (36)		
CAP score; mean ± SD, (n, %)		273.34 ± 65.02	282.18 ± 66.6	-1.1	0.276
	No significant steatosis	40 (40)	79 (39.5)	4.92	0.197
	Mild	17 (17)	18 (9)		
	Moderate	2 (2)	7 (3.5)		
	Severe	41 (41)	96 (48)		
Fibro scan score; mean ± SD		7.2 ± 2.05	7.03 ± 2.16	0.57	0.507
Fibrosis (n, %)	Present	77 (77)	149 (74.5)	0.22	0.636
	Absent	23 (23)	51 (25.5)		
Fibrosis stage (n, %)	No significant fibrosis	28 (28)	68 (34)	2.71	0.45
	Mild	38 (38)	61 (30.5)		
	Significant	30 (30)	58 (29)		
	Advance	4 (4)	13 (6.5)		
Dyslipidemia (n, %)	Normolipidemic	9 (9)	14 (7)	0.19	0.964
	Combined hyperlipidemia	3 (3)	8 (4)		
	Hyperlipidemia	1 (1)	3 (1.5)		
	Dyslipidemia with MetS	26 (26)	46 (23)		
	Low HDL-C	59 (59)	125 (62.5)		
	Hypertriglyceridemia	2 (2)	4 (2)		

**Figure 1:**
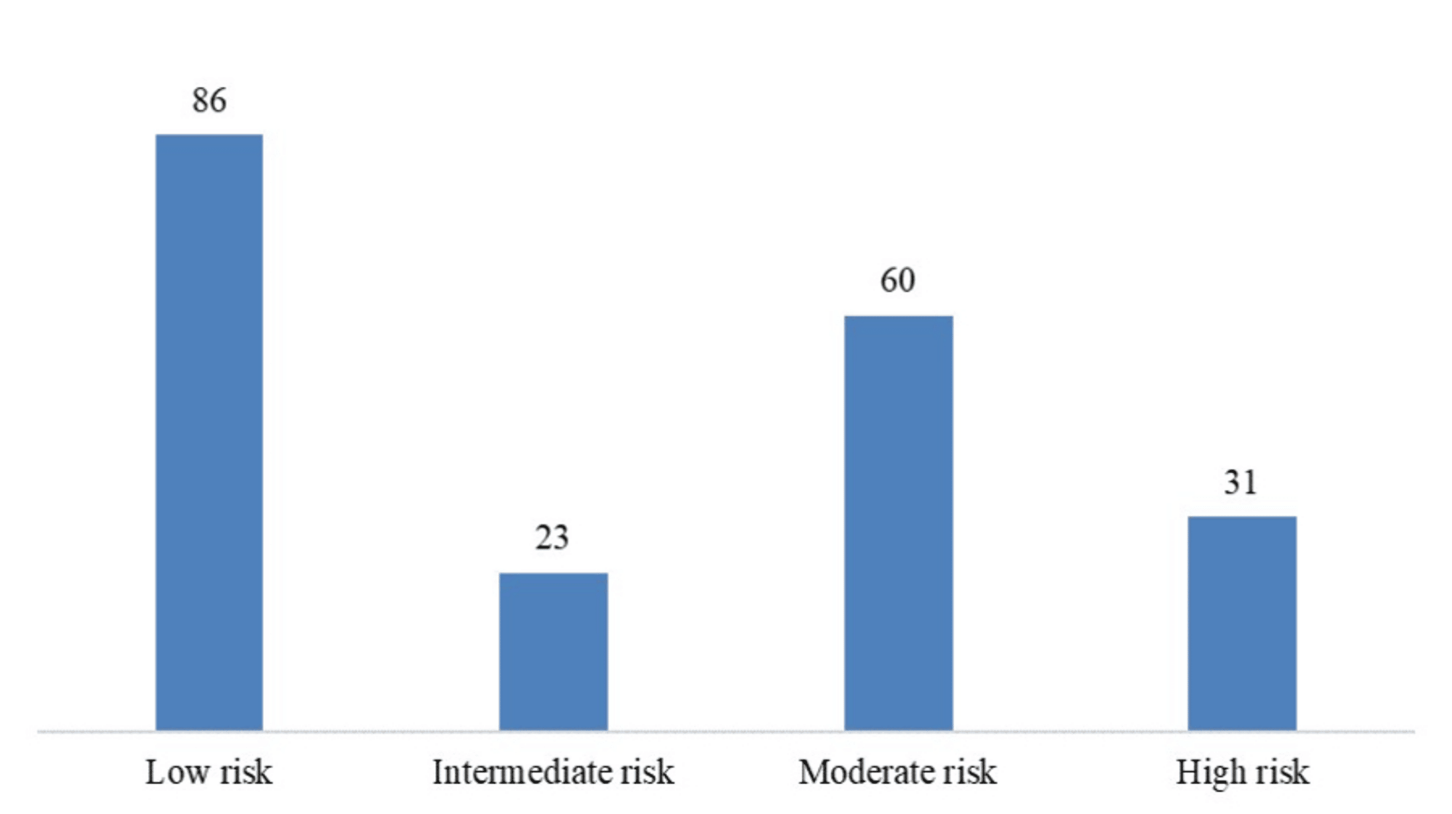
Distribution of ASCVD risk among controls.

The distribution of atherosclerotic cardiovascular disease (ASCVD) risk scores among controls showed that 43% of participants (n=86) were classified as low risk, 11.5% (n=23) as intermediate risk, 30% (n=60) as moderate risk, and 15.5% (n=31) as high risk. The overall mean ASCVD risk score in the study population was 10.53 ± 11.08, as shown in Figure 1. 

**Figure 2:**
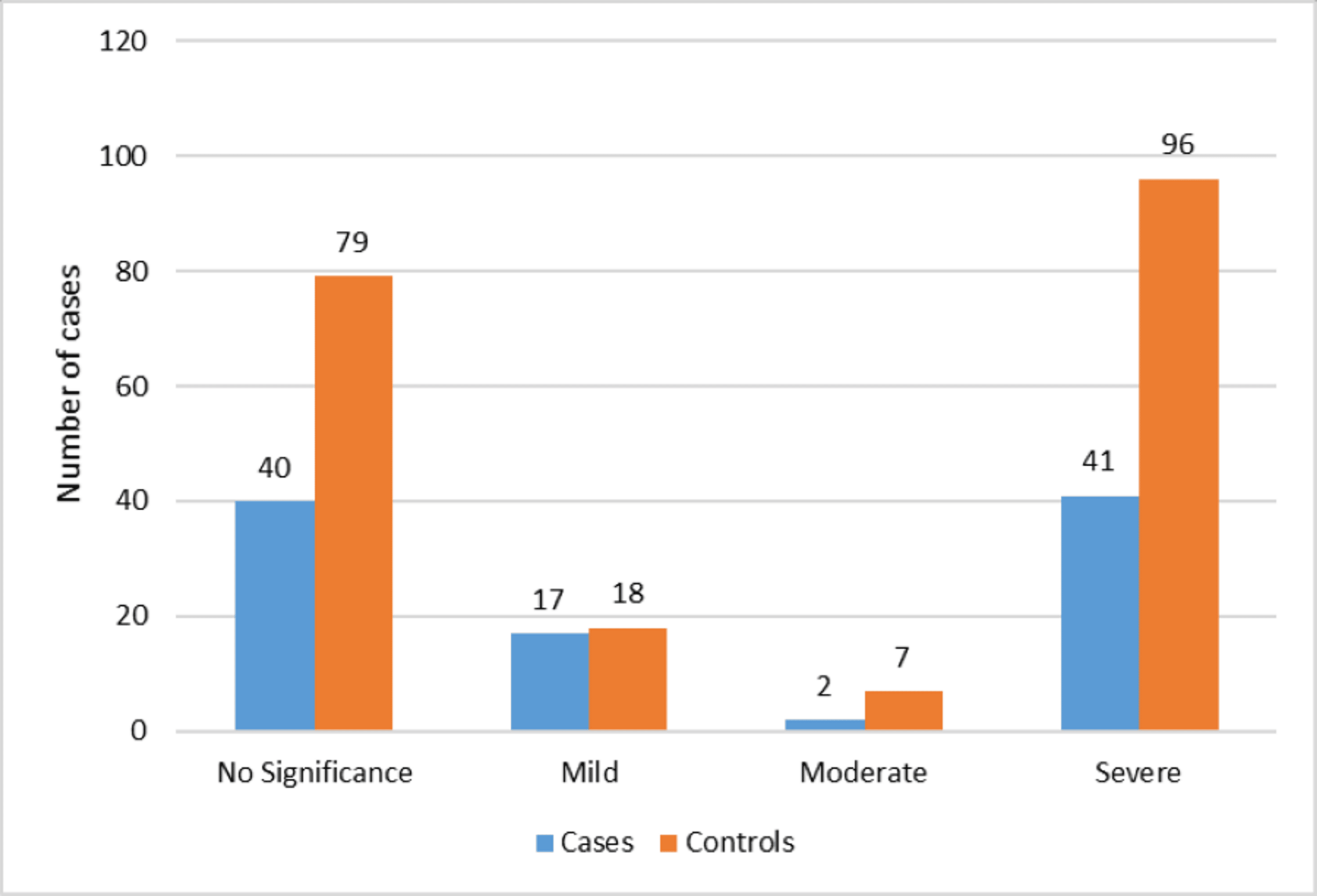
CAP severity among cases and controls. CAP: Controlled Attenuation Parameter.

Controlled attenuation parameter (CAP) measurements demonstrated that severe hepatic steatosis was the most common category, observed in 41 cases and 96 controls. Moderate steatosis was relatively uncommon, with only two cases and seven controls affected. Mild steatosis was detected in 17 cases and 18 controls, while 40 cases and 79 controls had no significant steatosis. The overall distribution of steatosis severity did not differ significantly between the two groups (Figure 2).

**Figure 3:**
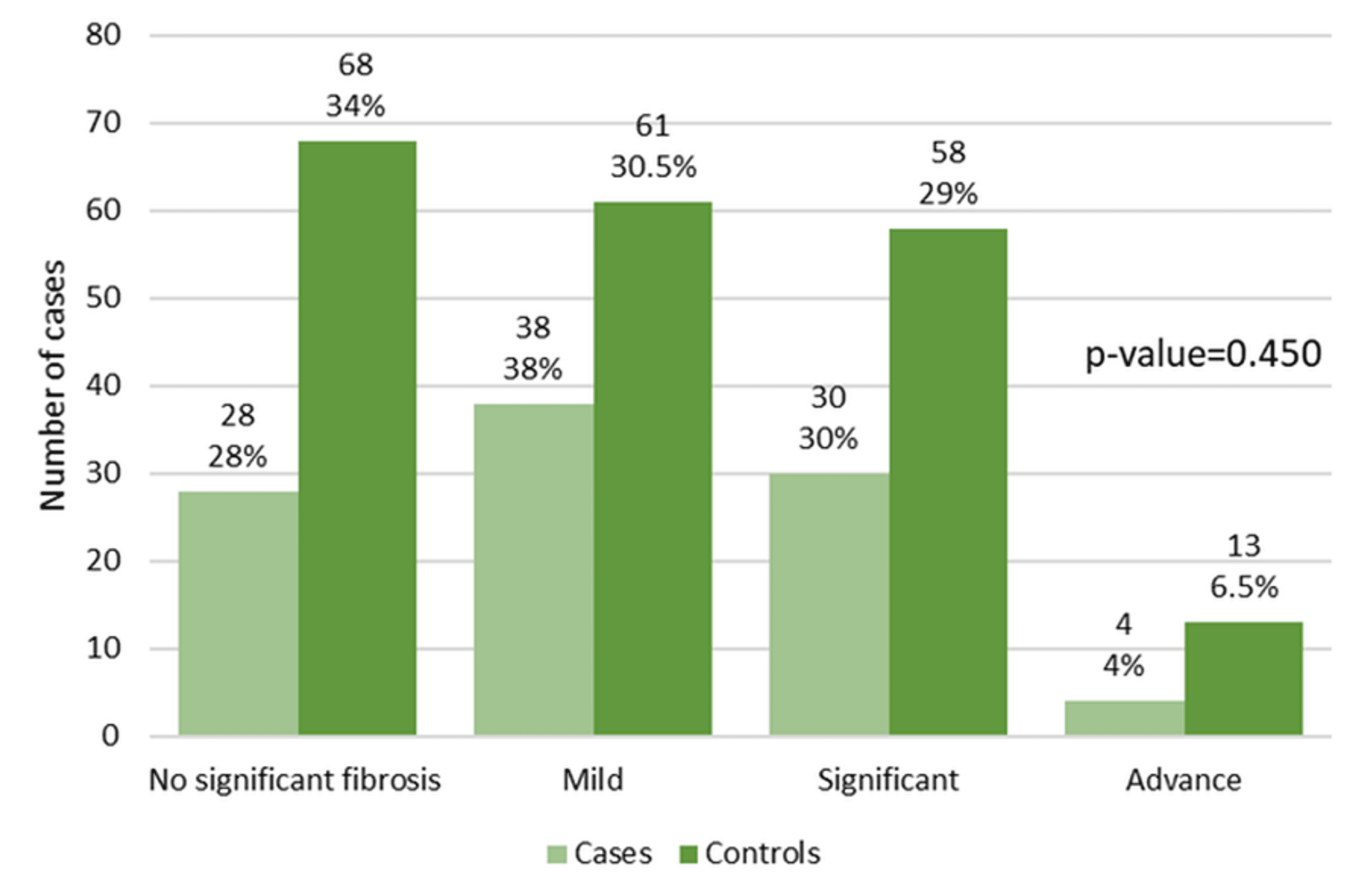
Fibrosis severity (FibroScan) among cases and controls

Among patients with NAFLD, the distribution of fibrosis severity was broadly similar between those with ischemic heart disease (cases) and those without (controls). No significant fibrosis was observed in 28 cases and 68 controls, while mild fibrosis was present in 38 cases and 61 controls. Significant fibrosis was documented in 30 cases and 58 controls, and advanced fibrosis in 4 cases and 13 controls. Overall, no statistically significant difference in fibrosis stage was detected between the two groups (Figure 3).

Binary logistic regression showed that most demographic, metabolic, and hepatic factors were not significantly associated with ischemic heart disease (IHD). Male gender and younger age (≤45 years) showed higher odds of IHD, but without statistical significance. Among hepatic parameters, a steatosis score of 0 was associated with lower odds of IHD (OR 0.52, p=0.048), while mild CAP severity was linked to higher odds (OR 2.21, p=0.040). No significant associations were observed for lipid ratios, fibrosis stage, or obesity (Table 2).

**Table 2:** Binary logistic regression analysis determining factors associated with ischemic heart disease in patients with NAFLD. Binary logistic regression was applied. *p-value≤0.05 considered as significant.

Parameters		OR (95% CI)	p-value
Gender	Male	1.157(0.710-1.886)	0.559
	Female	1.000	
Age Group	≤45 years	1.573(0.936-2.645)	0.087
	>45 years	1.000	
TG and HDL ratio	≤2	2.250(0.130-39.053)	0.578
	2.1 to 4.0	1.177(0.561-2.468)	0.666
	4.1 to 6.0	1.018(0.427-2.428)	0.968
	>6.0	1.000	
LDL and HDL ratio	≤2.9	1.043(0.637-1.708)	0.867
	>2.9	1.000	
Cholesterol and HDL ratio	≤3.5	1.024(0.407-2.574)	0.960
	3.51 to 5.0	1.222(0.605-2.470)	0.576
	5.1 to 9.0	0.869(0.395-1.912)	0.727
	>9.0	1.000	
Fibrosis	Present	1.146(0.652-2.014)	0.636
	Absent	1.000	
Hepatic Steatosis Score	0	0.517(0.270-0.993)	0.048*
	1	1.281(0.668-2.457)	0.456
	2	0.512(0.103-2.542)	0.413
	3	1.000	
Steatosis	Yes	0.957(0.579-1.582)	0.865
	No	1.000	
Dyslipidemia	Normolipidemic	1.286(0.194-8.534)	0.795
	Combined hyperlipidemia	0.750(0.087-6.468)	0.794
	Hyperlipidemia	0.667(0.039-11.285)	0.779
	Dyslipidemia with MetS	1.130(0.194-6.598)	0.892
	Low HDL-C	0.944(0.168-5.300)	0.948
	Hypertriglyceridemia	1.000	
Dyslipidemia with MetS	Yes	0.850(0.488-1.481)	0.566
	No	1.000	
CAP	No significant steatosis	1.186(0.699-2.009)	0.527
	Mild	2.211(1.037-4.715)	0.040*
	Moderate	0.669(0.133-3.358)	0.625
	Severe	1.000	
FibroScan	No significant fibrosis	1.338(0.401-4.461)	0.635
	Mild	2.025(0.615-6.666)	0.246
	Significant	1.681(0.504-5.605)	0.398
	Advance	1.000	
Obesity	Yes	0.495(0.069-3.566)	0.485
	No	1.000	

## Discussion

This study explored the relationship between non-alcoholic fatty liver disease (NAFLD) severity, assessed using FibroScan, and ischemic heart disease (IHD). Baseline demographic, anthropometric, and biochemical characteristics were broadly similar between the two groups, with the exception of lower HbA1c values in patients with IHD, suggesting better glycemic control in this subgroup. Importantly, no significant differences in FibroScan or CAP scores were observed between patients with and without IHD.

Previous evidence consistently demonstrates that NAFLD is independently associated with increased cardiovascular morbidity and mortality, with fibrosis emerging as the strongest predictor of adverse outcomes [7]. However, the lack of significant differences in hepatic parameters between groups in our cohort suggests that liver disease severity alone may not fully explain cardiovascular risk. Studies have shown that CAP scores correlate positively with coronary complexity, measured by SYNTAX scores, and that hepatic steatosis is linked to major adverse cardiovascular events (MACE) regardless of coronary artery disease extent [14,15]. In diabetic populations with IHD, NAFLD prevalence has been reported as high as 71%, with moderate steatosis associated with greater carotid intima-media thickness and poor glycemic control [16]. Conversely, some paradoxical findings have been reported, where steatosis was associated with lower all-cause mortality and reduced decompensation risk in patients with metabolic dysfunction-associated steatotic liver disease (MASLD) [17]. These heterogeneous findings suggest that the prognostic role of steatosis may vary by population and disease context.

Fibrosis severity, however, consistently shows stronger associations with cardiovascular outcomes. Recent studies demonstrate significant correlations between advanced fibrosis and elevated atherosclerotic cardiovascular disease (ASCVD) risk scores [18], as well as with established risk prediction models and vascular imaging markers [19]. Fibrosis stage F3 or greater has been independently associated with traditional cardiometabolic risk factors such as age, BMI, dyslipidemia, and hypertension [20]. Large cohort analyses also confirm a dose-response relationship, with intermediate and advanced fibrosis conferring progressively higher odds of cardiovascular disease [21]. Collectively, these findings support the growing recognition of liver fibrosis as a key driver of cardiovascular risk in NAFLD patients.

In our cohort, the ASCVD risk score distribution reinforced the overlap between metabolic and cardiovascular disease. Notably, even in the absence of IHD, nearly half of the controls were classified in the moderate-to-high risk range. This aligns with prior reports demonstrating that NAFLD patients often harbor elevated cardiovascular risk independent of liver disease severity [22,23]. Thus, while FibroScan and CAP remain valuable non-invasive tools for hepatic risk stratification, their integration with cardiometabolic profiling is essential for comprehensive assessment.

This study has limitations. Its cross-sectional design precludes causal inference, and results from a single-center cohort may not be generalizable. Because participants were recruited through consecutive sampling with a 1:2 case-to-control ratio from a tertiary-care facility, the sample may overrepresent individuals with metabolic comorbidities, introducing potential selection bias. FibroScan, although validated, may be less reliable in individuals with extreme obesity. In addition, unmeasured confounding factors cannot be excluded. Despite these limitations, our findings highlight the importance of adopting an integrated approach that combines hepatic and cardiovascular evaluation in patients with NAFLD.

## Conclusions

The severity of NAFLD, as measured by FibroScan, did not differ significantly between patients with and without IHD. However, an association between mild steatosis (CAP) and IHD was observed, suggesting that early hepatic changes may reflect cardiovascular risk. These findings emphasize the need to incorporate liver assessment into comprehensive cardiovascular risk evaluation. Future multicenter, longitudinal studies are warranted to clarify causal pathways and to determine whether non-invasive hepatic assessment can enhance cardiovascular risk prediction and management in NAFLD populations.
